# AIRE illuminates the feature of medullary thymic epithelial cells in thymic carcinoma

**DOI:** 10.1002/cam4.5777

**Published:** 2023-03-13

**Authors:** Minoru Matsumoto, Takuya Ohmura, Yuto Hanibuchi, Mayuko Ichimura‐Shimizu, Yasuyo Saijo, Hirohisa Ogawa, Ryuichiro Miyazawa, Junko Morimoto, Koichi Tsuneyama, Mitsuru Matsumoto, Takeshi Oya

**Affiliations:** ^1^ Department of Molecular Pathology Tokushima University Graduate School of Biomedical Sciences Tokushima Japan; ^2^ Division of Molecular Immunology Institute for Enzyme Research, Tokushima University Tokushima Japan; ^3^ Biozentrum University of Basel Basel Switzerland; ^4^ Student Lab, Tokushima University Faculty of Medicine Tokushima Japan; ^5^ Department of Pathology and Laboratory Medicine Tokushima University Graduate School of Biomedical Sciences Tokushima Japan

**Keywords:** AIRE, bioinformatics, single‐cell RNA‐seq, The Cancer Genome Atlas, thymic carcinoma, thymic epithelial tumor

## Abstract

Despite the clear distinction between cortical (cTECs) and medullary thymic epithelial cells (mTECs) in physiology, the cell of origin of thymic carcinomas (TCs) and other thymic epithelial tumors remained enigmatic. We addressed this issue by focusing on AIRE, an mTEC‐specific transcriptional regulator that is required for immunological self‐tolerance. We found that a large proportion of TCs expressed AIRE with typical nuclear dot morphology by immunohistochemistry. AIRE expression in TCs was supported by the RNA‐seq data in the TCGA‐THYM database. Furthermore, our bioinformatics approach to the recent single‐cell RNA‐seq data on human thymi has revealed that TCs hold molecular characteristics of multiple mTEC subpopulations. In contrast, TCs lacked the gene signatures for cTECs. We propose that TCs are tumors derived from mTECs.

## Graphical abstract

Thymic epithelial tumors originating from thymic epithelial cells (TECs) are classified into six types of thymomas (Type A, AB, B1‐B3 thymomas, and micronodular thymoma with lymphoid stroma [MNT]) and thymic carcinoma (TC). Despite the clear distinction between cortical (cTECs) and medullary TECs (mTECs) in physiology, the cell of origin of each TET has remained enigmatic. In the current study, we confirmed the expression of AIRE, an mTEC‐specific transcriptional regulator, in most TCs at both protein and mRNA levels. Furthermore, our bioinformatics approach has revealed that TCs hold molecular characteristics of multiple mTEC subpopulations, suggesting their cell of origin as mTECs.

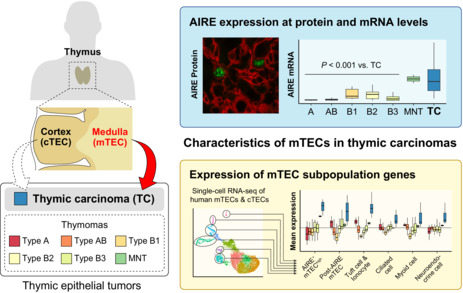

## 1 INTRODUCTION

Thymic epithelial tumors (TETs) originating from thymic epithelial cells (TECs) are classified into six types of thymomas (Type A, AB, B1–B3 thymomas, and micronodular thymoma with lymphoid stroma [MNT]) and thymic carcinoma (TC).^1^ In physiology, TECs consist of cortical (cTECs) and medullary TECs (mTECs), which play essential roles in the positive and negative selection of developing thymocytes, respectively.^2^ Despite this clear distinction, the cell of origin of each TET has remained enigmatic. This issue has been further hampered by the absence of organotypic appearance in TCs. To answer this question, we considered that investigation of AIRE expression in TCs might give us an important clue because the expression of AIRE, the mutation of which is responsible for the development of autoimmune polyendocrine syndrome type 1 (APS‐1),^3^ is unique to mTECs. AIRE exists in the mTECs as nuclear dots, and it promotes the expression of a broad spectrum of self‐antigens to establish self‐tolerance.^4^ In this regard, one study reported that AIRE was not detected in any of the 10 TC samples by immunohistochemistry.^5^

Here, we revisited the AIRE expression from TCs and other TETs by immunohistochemistry using our in‐house anti‐AIRE antibody.^6^, ^7^ Unexpectedly, we detected AIRE expression from most TC samples, suggesting that TCs hold the characteristics of mTECs. Because The Cancer Genome Atlas (TCGA) Project has provided a genomic landscape of TETs^8^ and the recent advent of single‐cell RNA‐seq (scRNA‐seq) analysis has revealed the diversity of mTECs, we have discussed the origin of TCs from novel viewpoints.

## 2 MATERIALS AND METHODS

### 2.1 Tissue specimens

Ninety specimens diagnosed as TET were used for immunohistochemical analyses (Table 1). While we prioritized the surgical specimen of the primary tumor, we also employed specimens from the secondary site if the primary tumor was not available. All tissue samples were obtained at Tokushima University Hospital, Tokushima, Japan.

**TABLE 1:** Detection of AIRE with immunohistochemical analysis in TETs.

Histology	*N*	Positive (%)	Negative (%)	MG in AIRE^+^ cases (%)	MG in AIRE^−^ cases (%)
A	8	0 (0)	8 (100)		0 (0)
AB	22	11 (50)	11 (50)	2 (18.2)	1 (9.1)
B1	8	8 (100)	0 (0)	4 (50)	
B2	24	20 (83.3)	4 (16.7)	8 (40)	1 (25)
B3	8	3 (37.5)	5 (62.5)	1 (33.3)	2 (40)
MNT	4	1 (25)	3 (75)	0 (0)	1 (33.3)
TC	16	12 (75)	4 (25)	0 (0)	0 (0)

Abbreviations: A, type A thymoma; AB, type AB thymoma; B1, type B1 thymoma; B2, type B2 thymoma; B3, type B3 thymoma; MG, myasthenia gravis; MNT, micronodular thymoma with lymphoid stroma; TC, thymic carcinoma.

### 2.2 Immunohistochemistry

Immunohistochemical analyses were performed using formalin‐fixed paraffin‐embedded specimens. Antigen retrieval was performed by autoclaving the samples in citrate buffer (pH 6.0) for 30 min. We used anti‐AIRE (polyclonal, 1:1000; see below) and anti‐cytokeratin AE1/AE3 (monoclonal, pre‐diluted, Nichirei) antibodies. Rabbit polyclonal anti‐human AIRE antibody was raised by immunizing the 13 amino acids residing between the PHD1 and PHD2 domains of human AIRE. Dako Autostainer Link 48 was used following the manufacturer's protocol. For immunofluorescence, we used secondary antibodies conjugated to Alexa Flour 594 (Invitrogen) or Alexa Flour 488 (Invitrogen).

### 2.3 Analysis of publicly available data

We utilized an RNA‐seq dataset from the TCGA Project archive (TCGA‐THYM). The TCGA‐THYM cohort was comprised of 108 thymomas (10 type A, 48 type AB, 12 type B1, 25 type B2, 11 type B3, and two MNT) and nine TCs. We used R package *edgeR* for the trimmed mean of M values (TMM) normalization.^9^ We reanalyzed scRNA‐seq data of human TECs from the GEO database under accession number GSE147520.^10^ The downstream analysis of scRNA‐seq data was conducted by R package *Seurat*.^11^

### 2.4 Statistics

Statistical significances of gene expression in the TCGA dataset (TC vs. other thymomas) were computed by one‐way ANOVA with post hoc Tukey HSD. Statistical significances of each gene expression in the scRNA‐seq data were computed by the Wilcoxon rank sum test. *p* value less than 0.05 was considered significant.

## 3 RESULTS

**FIGURE 1:**
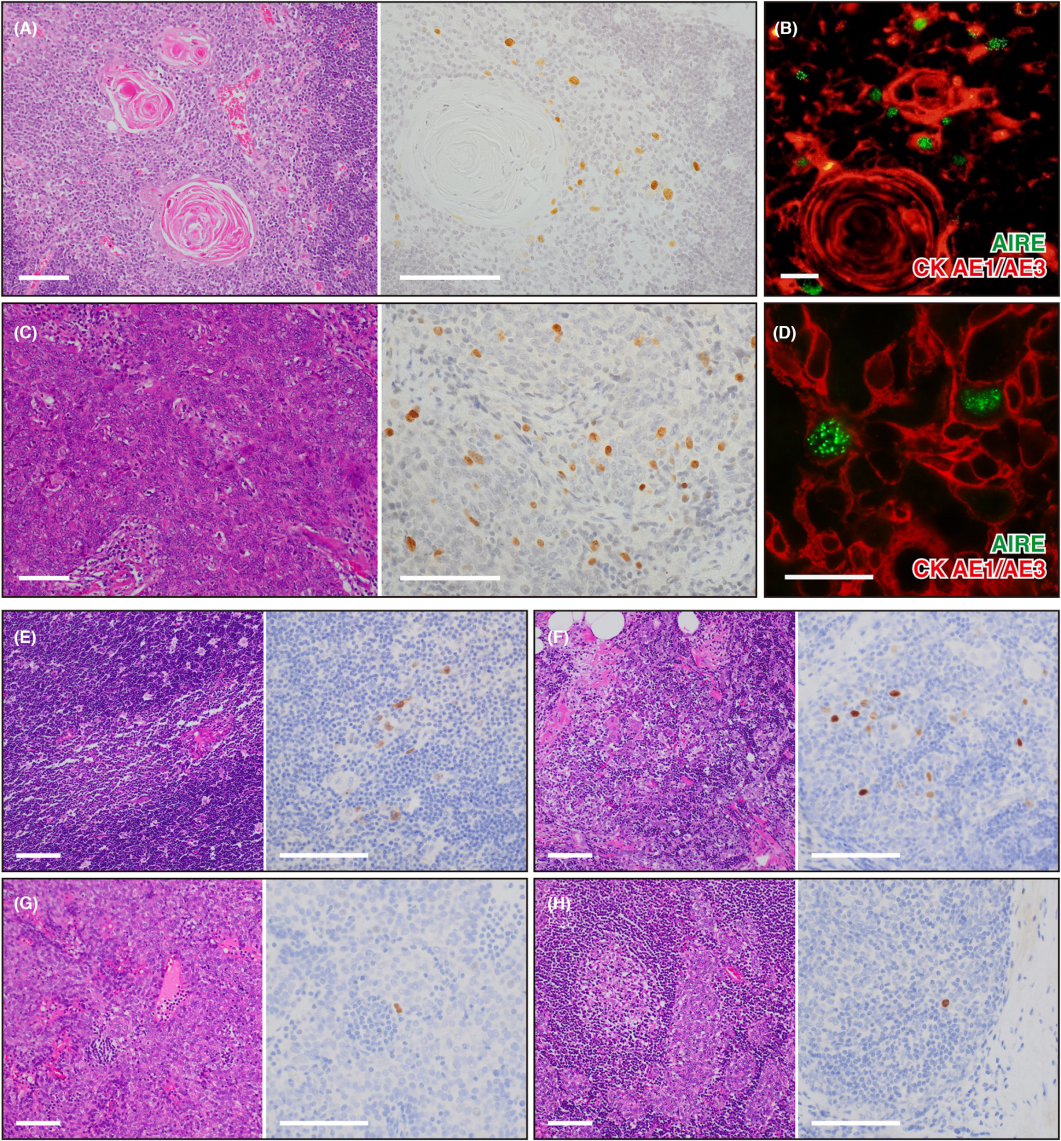
Expression of AIRE with nuclear dot morphology from thymic carcinomas.(A) Representative histology (left) and immunohistochemistry for AIRE (right) of the normal non‐neoplastic thymus. Scale bar, 100 μm. (B) Immunofluorescence for AIRE (green) and cytokeratin (CK) AE1/AE3 (red) of the normal non‐neoplastic thymus. Scale bar, 20 μm. (C) Representative histology (left) and immunohistochemistry for AIRE (right) of TC sample. Scale bar, 100 μm. (D) Immunofluorescence for AIRE (green) and CK AE1/AE3 (red) of TC sample. Scale bar, 20 μm. (E–H) Representative histology (left) and immunohistochemistry for AIRE (right) of type B1 thymoma (E), type B2 thymoma (F), type B3 thymoma (G), and micronodular thymoma with lymphoid stroma (H). Scale bar, 100 μm.

We examined a total of 90 samples diagnosed as TET (Table 1). We applied our in‐house polyclonal anti‐human AIRE antibody, whose sensitivity and specificity were strictly validated using the transgenic mice expressing human AIRE.^6^, ^7^ Staining of the normal human thymus (Figure 1A, left) with this antibody showed strong positivity for the nuclei of mTECs (Figure 1A, right). Under the fluorescence microscope, we observed AIRE expression as typical nuclear dots (Figure 1B).

All TC samples in our archive consisted of solid nests of atypical polygonal cells, as exemplified by Figure 1C, left. We found that 12 out of 16 TCs showed positivity for the anti‐AIRE antibody in the nucleus (Figure 1C, right and Table 1). Of note, AIRE was not expressed in every tumor cell. Instead, AIRE^+^ cells were scattered throughout the tumor tissues like normal thymus. The proportion of AIRE^+^ cells was variable among the samples ranging from ~1% up to ~30%. By immunofluorescence, AIRE in TCs showed authentic nuclear dot morphology (Figure 1D).

We found that all type B1 thymomas (eight out of eight) contained a few AIRE^+^ cells scattered in the area showing medullary differentiation (Figure 1 E). Type B2 (20 out of 24) and type B3 thymomas (three out of eight) also showed focal but intense AIRE staining, suggesting that a high proportion of type B thymomas contain the area showing differentiation to mTECs (Figure 1F,G). Type AB thymomas also showed scattered AIRE^+^ cells in type B‐like components, although at a slightly lower frequency (11 out of 22) compared with the conventional type B thymomas (Table 1). In contrast, AIRE was not detectable in the spindle‐shaped tumor cells in type A and AB thymomas. Interestingly, one MNT showed an unusual staining pattern with a single clear AIRE^+^ cell (Figure 1H). Overall, we did not see a correlation between the incidence of myasthenia gravis (MG) and the expression of AIRE in thymomas (Table 1). None of the patients with TCs developed MG in our cases.

**FIGURE 2:**
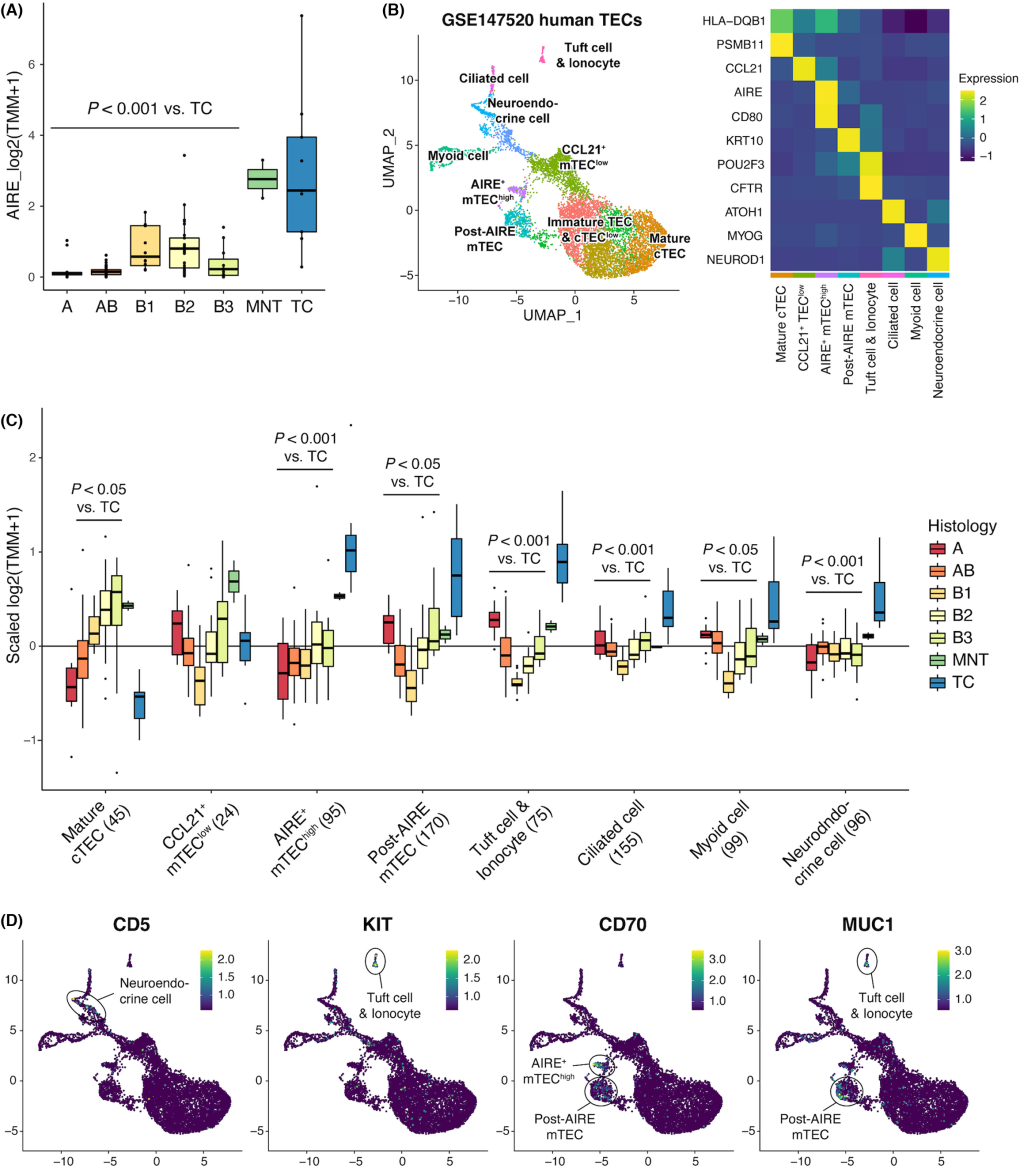
The bioinformatics approach uncovered the molecular characteristics of mTECs in thymic carcinomas. (A) Expression of human AIRE mRNA in the dataset from TCGA‐THYM. The whiskers of boxplots extend 1.5 times the interquartile range (IQR) from the top and bottom of the box. (B) Uniform Manifold Approximation and Projection (UMAP) visualization of the scRNA‐seq data of human TECs (left) and heatmap visualization of the expression of cluster‐characterizing genes (right). The data were extracted from the GEO database under accession number GSE147520. (C) Mean expression value of TEC subpopulation genes in bulk RNA‐seq data from the TCGA‐THYM dataset. The genes characterizing each subpopulation were identified by calculating statistical significances among the clusters (log_2_FC > 1, adjusted *p* < 0.05). The number in the brackets below the subpopulation name represents the number of genes employed for the analysis. The whiskers of boxplots extend 1.5 times the IQR from the top and bottom of the box. (D) Expression of *CD5*, *KIT*, *CD70*, and *MUC1* in scRNA‐seq data from human TECs.

To confirm the AIRE expression in TCs and other thymomas, we utilized RNA‐seq data from TCGA‐THYM.^8^ After redefining the histology, we used RNA‐seq data from nine TCs and 108 thymomas (10 type A, 48 type AB, 12 type B1, 25 type B2, 11 type B3, and two MNT) (Table S1). We detected the highest level of *AIRE* expression from TCs among TETs, and type B thymomas showed a lower but significant *AIRE* expression (Figure 2A). Two MNT samples showed high *AIRE* expression, consistent with the formation of Hassall's corpuscles in the digital slide.

We further extended our view using the recent data obtained by scRNA‐seq. scRNA‐seq has revealed the existence of multiple subpopulations for mTECs as exemplified by the mTECs reminiscent of tuft cells in the gastrointestinal tract.^10^, ^12^, ^13^ We reanalyzed and applied the scRNA‐seq dataset of human TECs to know the cellular characteristics of TCs more in depth.^10^ We identified multiple TEC subpopulations including mature cTEC, CCL21^+^ immature mTECs (mTEC^low^), AIRE^+^ mature mTECs (mTEC^high^), and post‐AIRE mTECs (Figure 2B). We also detected unique mTEC subpopulations such as myoid cells, neuroendocrine cells, ciliated cells, and thymic tuft cells (overlapping with ionocytes), consistent with the original report.^10^ We then extracted a group of genes that characterize each subpopulation. When we plotted the mean expression values of these genes onto TCGA‐THYM RNA‐seq data, we found that the expression of genes representing AIRE^+^ mTEC^high^ was the highest in TCs (Figure 2C, third left). Furthermore, TCs also showed higher expression of genes unique to diverse mTEC subpopulations (i.e., post‐AIRE mTECs, tuft cells/ionocytes, ciliated cells, myoid cells, and neuroendocrine cells) compared with other TETs, except for CCL21^+^ mTEC^low^ genes (Figure 2C). In contrast, the expression of genes characterizing cTECs was significantly low. Taken together, TCs hold the feature of mTECs consisting of various subpopulations and stages of differentiation. Finally, we noticed that the expression of molecules with diagnostic values for TCs (*CD5*, *KIT*, *CD70*, and *MUC1*)^14^, ^15^, ^16^, ^17^ was predominant in mTEC clusters (Figure 2D), further supporting our notion.

## 4 DISCUSSION

Our present study combining immunohistochemical analysis of AIRE with a bioinformatics approach has provided a novel insight into the pathobiology of TETs, especially for TCs. One possible reason for the discrepancy between the previous study^5^ and our present study regarding the detection of AIRE protein in TCs may be the difference in sensitivity of the antibodies utilized. RNA‐seq data in TCGA‐THYM were consistent with our results. Because TCs expressed multiple mTEC subcluster genes while lacking the cTEC gene signature, we consider that TCs are derived from the cells that have already undergone the lineage decision toward mTECs. A previous report demonstrating a tuft cell‐like gene signature in TCs^18^ further supported our notion. In this regard, the expression of CCL21^+^ mTEC^low^ genes was not high in TCs. Instead, it was relatively high in type A thymoma, a subtype historically considered to reflect the characteristics of mTECs. We consider that TCs and type A thymomas are derived from different subpopulations and/or developmental stages of mTECs; the former is closely associated with mature mTECs, whereas the latter is with immature mTECs.

Our findings have raised a new possibility to the important question: Why do we seldom see the development of autoimmunity in patients with TCs in contrast to thymomas? The absence of AIRE expression from most thymomas has been discussed with the development of MG.^19^ However, because both TCs (in which MG rarely develops) and type B thymomas (in which MG develops at the highest frequency) expressed AIRE at a rather high frequency by our immunohistochemical analysis, some other factors than the AIRE might be responsible for the development of MG in thymomas. Given that autoreactive T cells are produced as a result of the positive selection in the cortex in the first place, it may be intriguing to hypothesize that the absence of cTEC gene signatures in TCs may account for the weak association with autoimmunity.

In conclusion, our study has demonstrated that TCs hold the characteristics of mTECs with AIRE expression, suggesting their cell of origin as mTECs. However, expression profiling of TCs at single‐cell resolution is yet to be approached because our study is based on bulk RNA‐seq analysis. How our findings revealing the immunopathology of TCs can be utilized for the novel therapeutic strategy for this devastating tumor also awaits further study.

## AUTHOR CONTRIBUTIONS

**Minoru Matsumoto:** Conceptualization (equal); formal analysis (equal); investigation (equal); writing – original draft (equal). **Takuya Ohmura:** Data curation (equal); software (equal); visualization (equal). **Yuto Hanibuchi:** Investigation; visualization. **Mayuko Ichimura‐Shimizu:** Validation (equal). **Yasuyo Saijo:** Investigation. **Hirohisa Ogawa:** Supervision (equal); validation. **Ryuichiro Miyazawa:** Supervision (equal). **Junko Morimoto:** Supervision (equal). **Koichi Tsuneyama:** Supervision (equal); validation. **Mitsuru Matsumoto:** Resources (equal); writing – review and editing (equal). **Takeshi Oya:** Conceptualization; validation; writing – review and editing (equal).

## FUNDING INFORMATION

This work was supported in part by Tokushima University President.

## CONFLICT OF INTEREST STATEMENT

All the authors declare no conflict of interest.

## ETHICS STATEMENT

The present study was approved by the Ethics Committee of Tokushima University Hospital. They also waived the requirement to obtain written informed consent from patients, given the study's retrospective nature (approval number: 4107).

## Supporting information


**Table S1.** The results of the re‐definition of the TCGA‐THYM samples

## Data Availability

Data sharing is not applicable to this article as no new data were created or analyzed in this study.
